# Previously published drug interaction models predict loss of response for transoesophageal echocardiography sedation well but not response to oesophageal instrumentation

**DOI:** 10.1038/s41598-019-40366-3

**Published:** 2019-03-07

**Authors:** Fu-Wei Su, Chien-Kun Ting, Jing-Yang Liou, Yi-Chang Chen, Mei-Yung Tsou, Shen-Chih Wang

**Affiliations:** 1 Department of Anesthesiology, Taipei Veterans General Hospital, No. 201, Sec. 2, Shipai Road, Beitou District, Taipei City, 11217 Taiwan Republic of China; 2 National Yang-Ming University and School of Medicine, No. 155, Sec. 2, Linong Street, Taipei City, 112 Taiwan Republic of China; 3 Biomedical Technology and Device Research Laboratories, Industrial Technology Research Institute, Hsinchu, 195, Sec. 4, Chung Hsing Road, Chutung, Hsinchu, 300 Taiwan Republic of China; 4 Institute of Molecular Medicine and Bioengineering, National Chiao Tung University, Hsinchu, Taiwan, 1001 University Road, Hsinchu, Taiwan 300 Republic of China; 5 Department of Biological Science and Technology, Institute of Bioinformatics and Systems Biology, National Chiao Tung University, 1001 University Road, Hsinchu, 300 Taiwan Republic of China

## Abstract

Response surface models (RSMs) were used to predict effects of multiple drugs interactions. Our study was aimed to validate accuracy of the previous published volunteer models during transoesophageal echocardiography (TEE). This is a cross-sectional study with 20 patients scheduled for transesophageal echocardiography in Taipei Veterans General Hospital, Taiwan. Effect-site concentration pairs of alfentanil and propofol were recorded and converted to equivalent remifentanil and propofol effect-site concentrations. Observer’s Assessment of Alertness/Sedation (OAA/S) scores were assessed every 2 minutes. Using these data, previous published models of loss of response (LOR), intolerable ventilatory depression (IVD), and loss of response to esophageal instrumentation (LREI) were then estimated. Accuracy of prediction is assessed by calculating the difference between the true response and the model-predicted probability. Clinical events such as interruption of TEE were recorded. The average procedure time was 11 minutes. Accuracy for prediction of LOR and LREI is 63.6% and 38.5%, respectively. There were four patients experienced desaturation for less than 1 minute, which were not predicted by IVD model, and one interruption of TEE due to involuntary movement. The previous published drug-interaction RSMs predict LOR well but not LREI for TEE sedation. Further studies using response surface methodology are needed to improve quality for TEE sedation and clinical implementation.

## Introduction

Transoesophageal echocardiography (TEE), an important cardiovascular imaging modality, is widely used in hospitals^1^. In comparison to transthoracic echocardiography, TEE provides superior visualisation of the posterior cardiac structures and consequently provides additional diagnostic information. Owing to its semi-invasive nature, sedation is often required during TEE procedures^2^. However, the benefits of sedation during TEE are not well characterised^3^, and the methods for anaesthetic administration vary among medical centres^4–7^. In Taipei Veterans General Hospital, propofol in combination with alfentanil is used to sedate patients undergoing TEE. As compared to using propofol alone, the co-administration of opioid with propofol facilitates oesophageal instrumentation but it increases the risk of respiratory depression^8^. Thus, it is important to understand the pharmacodynamic interaction and dosage-effect relationship between propofol and alfentanil.

Response surface models (RSMs), unlike traditional isobolograms that represent the concentrations of combirned agents to produce a single degree of drug effect (*e.g*., a C50 level—the concentration producing 50% of maximal drug effect), characterise the complete spectrum of interaction between two or more agents for possible levels of concentration and effects in response to a given stimulus^9^. Several medical procedures requiring moderate sedation and analgesia have evaluated these published RSMs^10–12^. Using a modified Greco construct equation^13^, LaPierre *et al*.^14^ developed RSMs to investigate the effects of the combination of remifentanil and propofol in volunteers. By utilizing the Observer’s Assessment of Alertness/Sedation (OAA/S) scale (Table 1)^15^, the effects investigated included a loss of responsiveness (LOR), a loss of response to oesophageal instrumentation (LREI), and intolerable ventilatory depression (IVD). To our knowledge, no study has explored their application in clinical TEE sedation. In comparison to healthy volunteers, patient managements are usually complicated with aging and comorbidities. We hypothesised that there should be differences between predicted and real responses in clinical settings. Therefore, the aim of our study was to validate the accuracy, as our primary outcome measures, of the RSMs previously published by LaPierre *et al*.^14^ during TEE sedation. We also recorded clinical events such as interruption during TEE sedation for our secondary outcome measures. Our study could yield valuable information on how well these drug interaction models developed from human volunteers would predict pertinent events of TEE sedation.

**Table 1:** The Observer’s Assessment of Alertness/Sedation (OAA/S) scale.

Value	Description
5	Responds readily to name spoken in normal tone.
4	Lethargic response to name spoken in normal tone.
3	Responds only after name is called loudly and/or repeatedly for the individual to open their eyes.
2	Responds only after moderate prodding or shaking.
1	Does not respond to moderate prodding or shaking

^a^An OAA/S score of 1 is considered unresponsive.

## Material and Methods

### Patient selection

This study was approved by the Institutional Review Board of Taipei Veterans General Hospital, Taipei, Taiwan (IRB 2014-12-001BC). Informed consent was obtained from all patients. The research protocol was performed in accordance with relevant guidelines/regulations. Among the patients scheduled for TEE in 2016, 20 patients were recruited for this study. Exclusion criteria included those patients with decompensated heart failure, class IV heart failure status according to the New York Heart Association Functional Classification, hearing or verbal impairment, neurologic or behavioural disorders, habitual sedative use, and allergy to propofol or alfentanil. Baseline blood pressure, heart rate, sex, age, weight, and height were recorded for each patient enrolled in the study.

### Experimental protocol

No sedative medication was used before the TEE procedure. After arrival to the examination room, supplemental oxygen of 5 L/minute was initiated via a nasal cannula. A 22-gauge intravenous catheter was then inserted for drug administration. Each patient was administered topical anaesthetic spray, and standard monitoring comprised electrocardiography, pulse oximetry, Bispectral index (BIS) monitoring, and non-invasive blood pressure monitoring. Vital signs were recorded both manually and using a computer. Bolus intravenous doses of propofol and alfentanil were simultaneously administered by an experienced anaesthesiologist. Additional doses of propofol (10 to 15 mg) or alfentanil (200 to 300 μg) were administered at 2 minutes after the initial bolus dose if the patient was still able to respond to verbal commands [OAA/S > 2].

The TEE probe (S7-2 Omni III, Philips) was used, and procedures were performed by three experienced cardiologists. The TEE operators were aware of the patients’ vital signs but were blinded to the anaesthetic agents administered.

### Effect measures

The OAA/S scale was evaluated by an independent observer. After induction, the patient’s level of sedation was assessed every 2 minutes using the OAA/S scale beginning from the simultaneous bolus administration of propofol and alfentanil. The following 3 measures were evaluated: loss of responsiveness (LOR) defined as an OAA/S score of 1; intolerable ventilatory depression (IVD) defined as a consistent decrease in the oxygen saturation (SpO_2_ < 90%) levels measured using a pulse oximeter); and loss of response to oesophageal instrumentation (LREI) defined as the absence of gag reflex and voluntary or involuntary movements, and heart rate or arterial blood pressure changes from 20% of baseline values, which were recorded before instrumentation.

### Primary outcome measures: the accuracy of RSMs

Using the SigmaPlot version 12.5 software (Systat Software, Inc., San Jose, CA, USA) and the previously published model parameters used to estimate each of the effect measures (Table 2)^14^, the predicted probability of the patient response was calculated. Pharmacokinetic profiles, including effect-site concentrations of propofol and alfentanil, were estimated using the TIVA Trainer simulation program (version 8, Build5, Guttabv, EuroSIVA, The Netherlands). We used Maitre *et al*.’s model^16^ for alfentanil, whereas Schneider *et al*.’s model^17^ was used for propofol. Alfentanil effect-site concentrations were converted to remifentanil equivalents by using a remifentanil-to-alfentanil potency ratio of 19:1^18^.

**Table 2:** Interaction model parameters for loss of responsiveness, loss of response to oesophageal instrumentation, and intolerable ventilatory depression.

	C_50 remi_	C_50 prop_	N	α
LOR	33.1	2.2	5.0	3.6
LREI	9.8	3.8	3.7	4.5
IVD	4.1	7.0	3.2	3.0

Abbreviations: LOR, loss of responsiveness; LREI, loss of response to oesophageal instrumentation; IVD, intolerable ventilatory depression; C_50remi_ (ng/mL) and C_50prop_ (μg/mL) represent effect-site concentrations for each drug that produces 50% probability of the maximal effect; n is the slope of the pharmacodynamic response curve; and α is the extent of interaction between the remifentanil and propofol for a given drug effect.

^a^Interaction model parameters taken from LaPierre *et al*.^8^.

Two graphical approaches were used. Contour graphs derived from the observed responses and topographical rendering of the modified model predictions were drawn. A graphical representation of the modified model was created by plotting the 5%, 50%, and 95% isoboles representing the predicted propofol-alfentanil effect-site concentrations that produced an equivalent effect. The second plot presented a modified three-dimensional modified RSM prediction.

An assessment of the model predictions was made by calculating the percentage of accurate predictions. Accuracy of prediction was assessed by calculating the difference between the true response and the model-predicted probability. The model was considered “accurate” if the difference was < 0.5. The total percentage of the accurate predictions was also obtained. During this calculation, LOR was assigned with a value of 1 if the patient’s OAA/S score was 1, and 0 if the OAA/S score was 2, 3, 4, or 5. For example, if the LOR model predicted that there was a 0.7 probability that the OAA/S score was 1, and the observation yielded an OAA/S score of 1, then 1.0 − 0.7 = 0.3. An absolute difference < 0.5 was defined as accurate.

### Secondary outcome measures: clinical events

Clinical events of desaturation (SpO_2_ < 90%), additional anaesthetic agent administration, and interruption of TEE were recorded by an independent observer. Interruption of the procedure was defined as withdrawal of the TEE probe due to patient conditions. For desaturation persisting for more than 1 minute, the anaesthesiologist would alarm the TEE operator to remove the probe and initiate mask ventilation. Ineffective mask ventilation was further managed with a nasal airway. Additional propofol or alfentanil doses would be administered if the patient had spontaneous eye opening, painful facial expressions, cough, gag reflex, or involuntary movements during the procedure. Withdrawal of the TEE probe for such patient responses was at the discretion of the TEE operators.

After the procedure, the patient was transferred to the post-anaesthetic recovery unit after verbal arousal was possible. Patients were discharged after obtaining an OAA/S scale of 4. Before discharge, patients would be asked by the independent observer if they remembered anything during the TEE procedure.

## Results

Twenty patients with physical status ranging from class II to III of the American Society of Anesthesiologists (ASA) classification were enrolled, and all subjects completed the study.

Patient demographics and indications for TEE are presented in Table 3. The mean age of the participants was 62 years. The mean cumulative dose of propofol was 0.7 ± 0.3 mg/kg and that of alfentanil was 6.1 ± 2.0 μg/kg. There was no significant difference in age, BMI, blood pressure, and procedure time between the male and female patients. The mean examination time for all patients was 11 minutes. The most common indication for TEE was atrial fibrillation.

**Table 3:** Patient demographics.

Age, years	61.9 ± 12.2
Weight, kg	65.5 ± 11.1
Height, cm	162.0 ± 8.0
BMI, kg/m^2^	25.0 ± 3.3
Systolic blood pressure, mmHg	152.4 ± 20.8
Diastolic blood pressure, mmHg	89.8 ± 14.8
Procedure time, min	11.1 ± 3.8
Sex (male/female)	7/13
Indication for TEE	
Atrial fibrillation	13
Valvular heart disease	5
Others*	2
ASA class	
II	14
III	6

Abbreviations: BMI, body mass index; TEE, transoesophageal echocardiography; ASA, American Society of Anesthesiologists.

*One patient had atrial septal defect (ASD); another patient had pulmonary stenosis.

**Figure 1:**
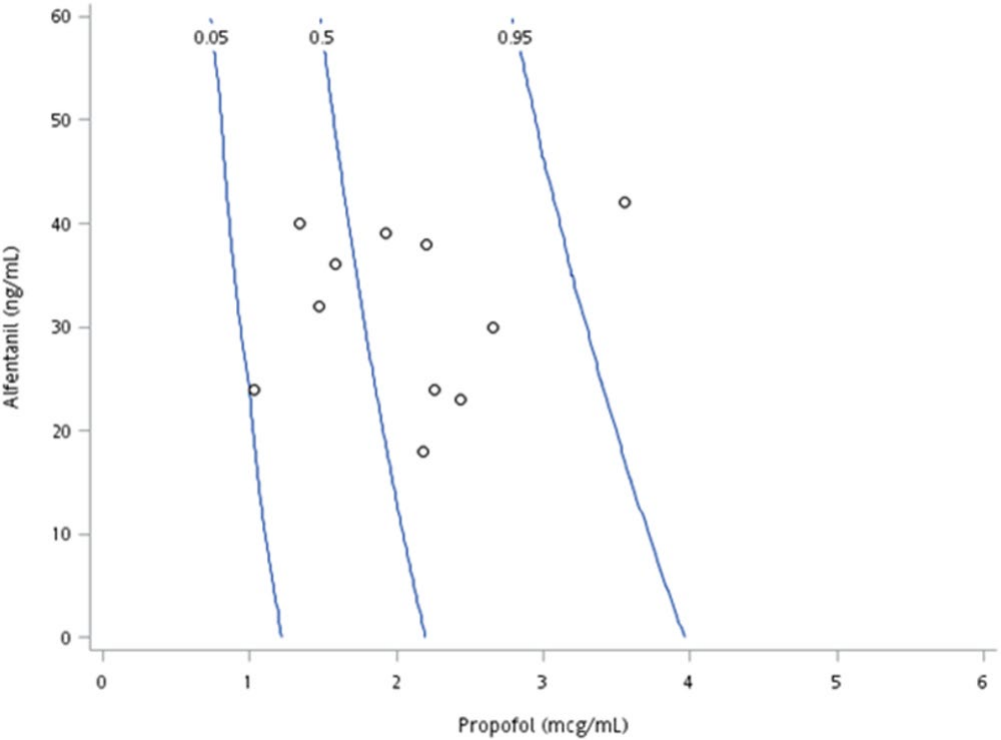
Contour graph of the modified loss of responsiveness (LOR) interaction model versus effect-site concentration. Solid lines represent the 5%, 50%, and 95% probabilities of OAA/S = 1 as predicted by the LOR interaction model.

The LOR interaction model predicting the probability that patients would have an OAA/S score of 1 at the onset of LOR is shown in Fig. 1. A total of 57 effect-site concentration pairs of propofol and alfentanil were available. Panel A presents the propofol-alfentanil effect-site concentrations at the onset of LOR (OAA/S = 1). The solid lines represent the 5%, 50%, and 95% probabilities of OAA/S = 1 (interaction model for LOR). A majority of the data points are above the 50% isobole; the accuracy rate for the prediction of OAA/S = 1 is 63.6%.

**Figure 2:**
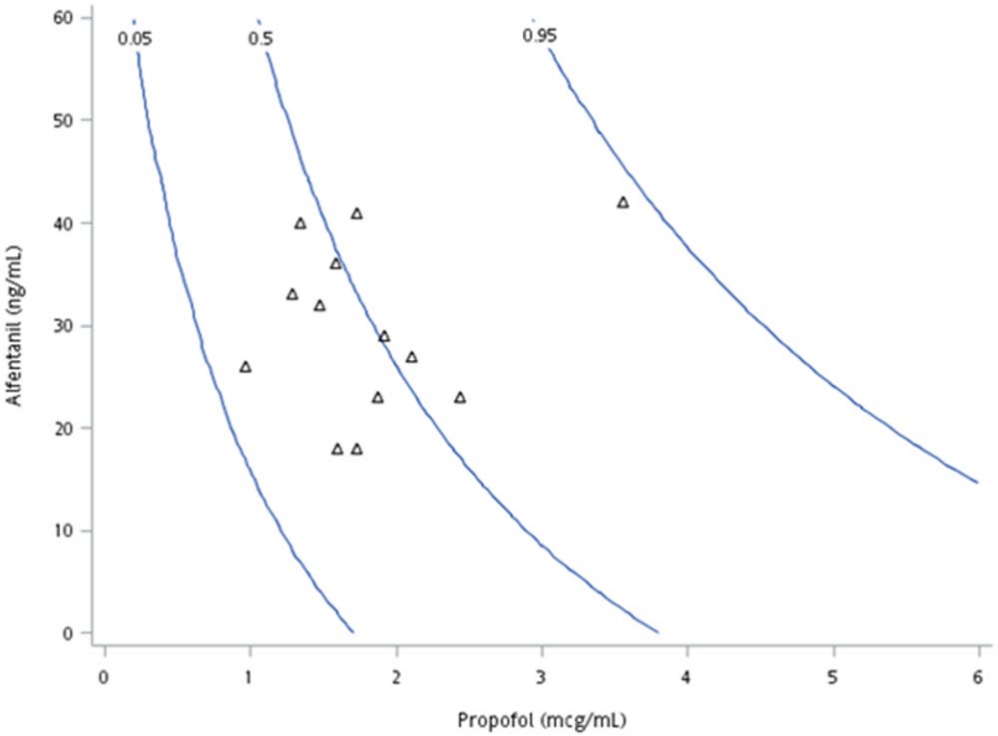
Contour graph of the modified loss of response to oesophageal instrumentation (LREI) model versus effect-site concentration. Solid lines represent the 5%, 50%, and 95% probabilities of no response to oesophageal instrumentation as predicted by the LREI interaction model.

Thirteen patients had LREI. Their propofol-alfentanil effect-site concentrations plotted with the 5%, 50%, and 95% isoboles from the LREI model are shown in Fig. 2. Among these patients, 5 demonstrated an LREI probability above the 50% isobole and an accuracy rate for the prediction of LREI of 38.5% (Panel A). Seven patients responded to EI with gag reflex, coughing, or involuntary movements.

In addition to validating the accuracy of previously published RSMs for LOR and LREI, we also validated the accuracy of IVD prediction. Of the four patients who exhibited IVD, the interaction model predictions for IVD revealed that all observations were below the 50% isobole (data not shown).

Clinical events are summarised in Table 4. Among the twenty patients, 12 were administered additional anaesthetic agents. The most common cause for additional anaesthetic agents was spontaneous eye opening. Four patients had desaturation for less than 1 minute and were managed with stimulation of jaw thrust only. There was only one interruption of TEE, and withdrawal of the probe was performed owing to involuntary movements. None of the patients complained of bad memories during the procedures.

**Table 4:** Clinical events during TEE sedation.

Additional anaesthetic agents	
Spontaneous eye opening	5
Involuntary movements	2
Cough	3
Gag reflex	2
Desaturation	4
Interruption of TEE	1

TEE, transoesophageal echocardiography.

**Figure 3:**
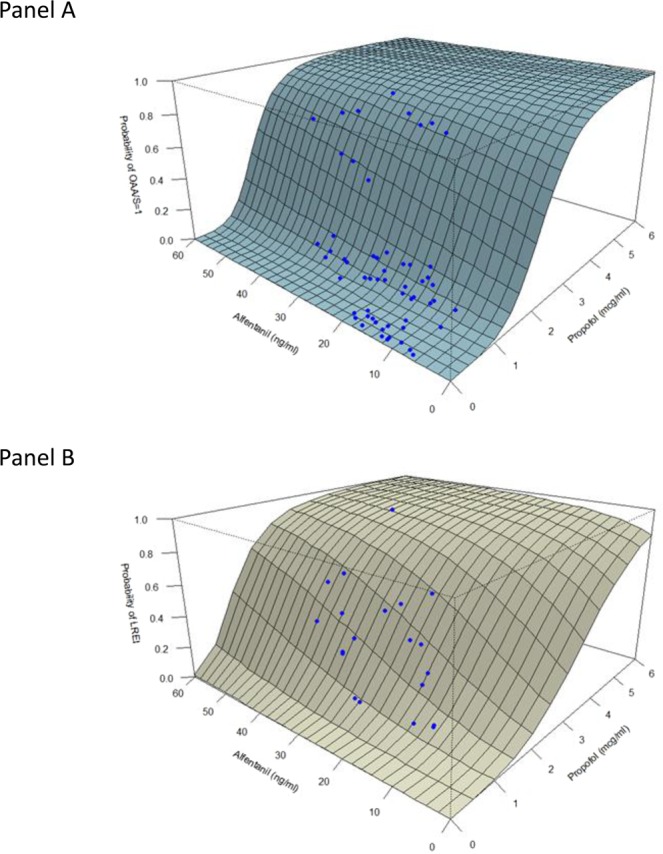
The modified response surface model for loss of responsiveness (LOR) (Panel A) and loss of response to oesophageal instrumentation (LREI) (Panel B) for propofol-alfentanil interaction between the two drugs. Each dot represents concentration pairs of our patients. OAA/S = Observer’s Assessment of Alertness and Sedation scale.

The RSM predictions for LOR and LREI, which are adapted from La Pierre *et al*.’s study are shown in Fig. 3A,B. Maitre *et al*.’s model^16^ was used to calculate alfentanil effect-site concentrations, which were converted to remifentanil equivalents by using a remifentanil-to-alfentanil potency ratio of 19:1^18^. Each dot on both panels represented one concentration pair in our clinical data. The graphs showed both models and allowed direct comparison of the drug interactions. Panel A presented a sharp but consistent probability decline when the surface approached the alfentanil axis. The effect was less pronounced in Panel B, indicating that the alfentanil effect was more prominent in producing LREI than LOR.

**Figure 4:**
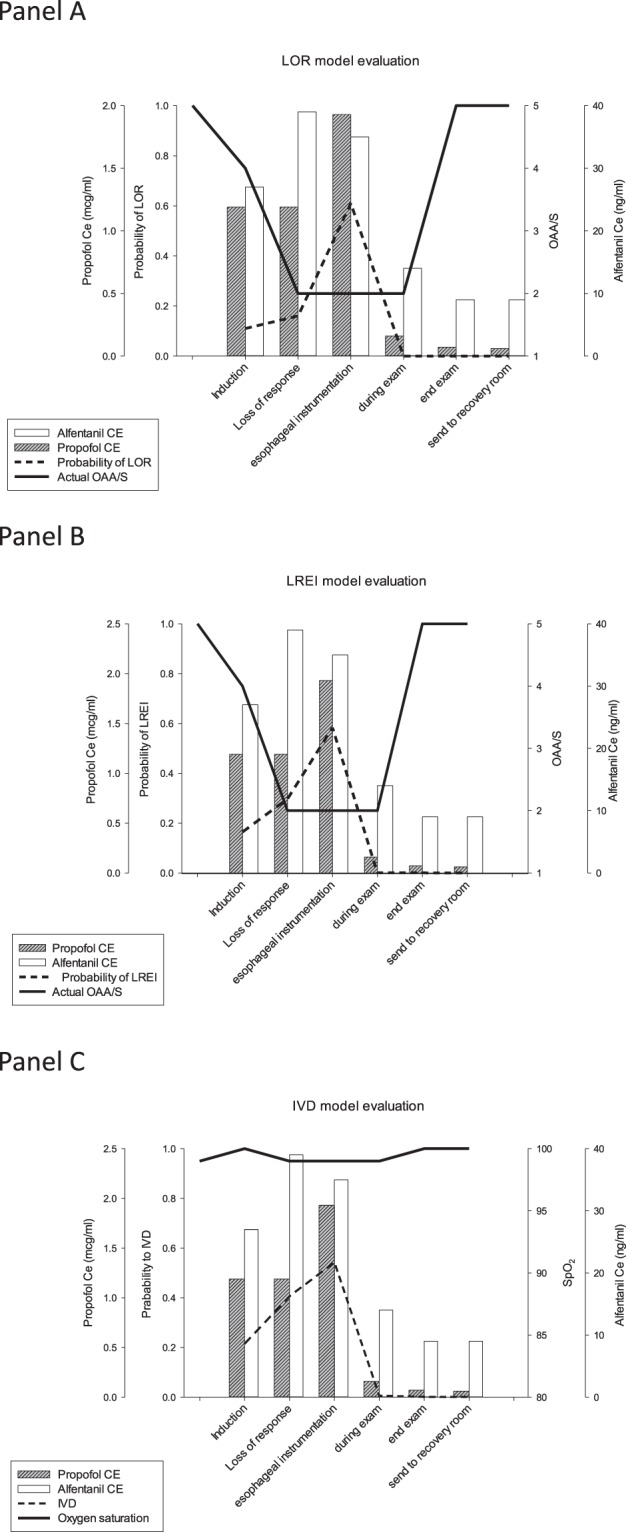
A sample patient’s predicted alfentanil and propofol effect-site concentrations alone with predictions of loss of responsiveness (LOR), loss of response to oesophageal instrumentation (LREI), and intolerable ventilatory depression (IVD) over time. Propofol and alfentanil effect-site concentrations are plotted in six events, namely induction, loss of response, oesophageal instrumentation, during examination, end of examination, and transfer of patient to the recovery room. For probabilities of LOR (Panel A) and loss of response to LREI (Panel B), the corresponding OAA/S scale is shown. For probabilities of IVD (Panel C), oxygen saturation (SpO_2_) changes are shown. OAA/S = Observer’s Assessment of Alertness and Sedation scale.

Figure 4 presented illustrations of one sample patient’s predicted alfentanil and propofol-effect site concentrations and probabilities of LOR (Panel A), LREI (Panel B) with OAA/S scale, and IVD (Panel C) with SpO_2_ during the TEE procedure. In this patient, the trend of OAA/S scale changes were reversely correlated with drug concentration pairs in LOR and LREI. However, in spite of the high probability of IVD (0.54) at oesophageal instrumentation, no desaturation was observed.

## Discussion

Our study revealed that healthy volunteer models may not fully emulate the complexities of the clinical environment, and the differences between the studied patients and their clinical demographics might impact model performance to some degree. The accuracy was further hampered under stressful condition such as oesophageal instrumentation.

Effect-site concentrations of propofol and alfentanil in our patients had limited ranges in comparison to those of LaPierre *et al*.’s patients as shown in Fig. 3. Drug concentration pairs in healthy volunteers with crisscross design might construct more comprehensive models. Therefore, we conducted a validation study to explore the practicability of the existing models instead of building a new set of RSMs using drug concentration pairs from our patients.

To the best of our knowledge, this is the first study that evaluated the accuracy of three RSMs (LOR, LREI, and IVD) in patients undergoing sedation for TEE procedures. We found that the LOR model predicted patient responsiveness better than the LREI model; however, the LREI model was able to predict responses to oesophageal instrumentation when none were observed in the LOR model. Specifically, this model indicated that higher alfentanil-propofol concentration pairs would be needed to block the response to oesophageal instrumentation than were actually needed. Moreover, the IVD model may be applied by clinicians to predict apnoea risk in sedated patients.

In terms of tolerance to probe insertion and manipulation during the TEE procedure, the LREI model showed lower accuracy and prediction rates. Mainly, nine of the thirteen patients had low probability in the model’s prediction whilst successfully achieving LREI. As compared to LaPierre *et al*.’s study participants^14^ (mean ± SD of age: 25 ± 4 years), our patients were older (mean ± SD of age: 62 ± 12 years). Elderly patients are more sensitive to propofol’s sedative and adverse effects^19^. As a consequence, our patients received lower propofol concentrations during the TEE procedure. LaPierre *et al*.’s study^14^ suggested a combination of high propofol (2–3 μg/mL) and low remifentanil (0.8 ng/mL) effect-site concentration to block the EI stimulus. However, despite the lower propofol effect-site concentration (1.25 ± 0.43 μg/mL) in our patients during the procedure, LREI was still observed. Notably, there was only one interruption of TEE examination and none of the patients complained of awareness during the procedure in our study. Moreover, the TEE probe diameter used in this study was 14.9 mm, whereas that in LaPierre *et al*.’s study [10] was 14 mm (blunt end bougie). These differences might explain partly the low accuracy rate for LREI prediction. Modification for LREI model to accommodate older subjects might be needed.

We had to convert alfentanil to remifentanil according to their potency before calculation for RSM’s accuracy. Our previous studies indicated that such conversion did not affect these RSMs’ applications to sedate patients undergoing medical procedures^10–12^.

In comparison to benzodiazepines, propofol provides rapid sedation and recovery with minimal lingering sedation-related side effects^20^ and greater post-procedure satisfaction^21^. Opioids are frequently used as adjuncts to offset patient discomfort^2^. Unfortunately, the risk for apnoea associated with dose titration limited the use of propofol with or without opioid for sedation by non-anaesthesiologists^2,8^. In our study, ventilation should be adequate as data of the majority of our patients fell below the 50% isobole in the IVD model. Anaesthetic agents were carefully titrated during our study to avoid unwanted side effects such as apnoea^22^. However, 4 patients still experienced desaturation but recovered within 1 minute without further management. Especially, these four inadequate ventilation episodes were not predicted by the IVD model. Our observations for IVD and LREI models consistently showed that lower propofol and alfentanil concentrations might reach adequate anaesthetic depth and even cause unwanted side effects in clinical settings. A large-scale clinical study is needed to investigate whether applying the IVD model can decrease the risk of apnoea in patients administered sedatives during TEE.

## Limitation

Several potential limitations of this study should be noted. First, our study was not designed to address the cost-effectiveness and safety of combined propofol and alfentanil administration for TEE sedation. Second, we only investigated the modified Greco model to describe the propofol-remifentanil interaction on the OAA/S scale, although there are several other available interaction model structures (i.e. logit, hierarchy, and so on)^23,24^. The clinical implications of using different interaction model structures for the predictions may warrant further investigation. Third, all RSMs used in this study were developed from younger volunteers with an average age of 24 years. We applied the modified models to our patients aged 30 to 80 years under the assumption that elderly patients would behave in a similar manner as young volunteers do. From the present study, the models seemed to be appropriate for our patients. However, further investigation on age limit for the adult model should be studied and justified. Fourth, all our patients received topical anaesthetic spray before the procedure; however, in the original study^14^, it seems that this procedure was not performed. Thus, this may have potentially affected the results.

## Conclusion

The previously published drug-interaction RSMs for upper gastrointestinal endoscopy can predict LOR but not LREI. Although desaturation was not predicted by the IVD model, none of the patients had interruption of TEE examination. This might indicate the practicability of the IVD model for clinicians in predicting apnoea in sedated patients. Further studies using the RSM are needed to improve the quality of TEE sedation and its clinical implementation.
